# *Origanum majorana* Attenuates Nephrotoxicity of Cisplatin Anticancer Drug through Ameliorating Oxidative Stress

**DOI:** 10.3390/nu8050264

**Published:** 2016-05-05

**Authors:** Amel M. Soliman, Shreen Desouky, Mohamed Marzouk, Amany A. Sayed

**Affiliations:** Zoology Department, Faculty of Science, Cairo University, Giza 12613, Egypt; soliman.amel5@gmail.com (A.M.S.); shereen_desokey@yahoo.com (S.D.); marzouk@sci.cu.edu.eg (M.M.)

**Keywords:** cisplatin, nephrotoxicity, *Originum majorana*, oxidative stress

## Abstract

Despite the fact that cisplatin is an important anticancer drug, its clinical utilization is limited by nephrotoxicity during long term medication. Combined cisplatin chemotherapy with plant extracts can diminish toxicity and enhance the antitumor efficacy of the drug. This study evaluated the effect of *Originum majorana* ethanolic extract (OMEE) on cisplatin-induced nephrotoxicity. Eighteen male rats were divided into three groups as follows: a control group, a group treated with cisplatin (3 mg/kg body weight), and a group that received both cisplatin and OMEE (500 mg/kg body weight) for 14 days. Cisplatin induced a significant increase in creatinine, urea, uric acid, blood urea nitrogen, malondialdehyde, and nitric oxide levels. However, glutathione, superoxide dismutase, and catalase levels were significantly diminished. Conversely, OMEE significantly modulated the renal and oxidative markers negatively impacted by cisplatin. OMEE significantly reduced the effects of cisplatin-induced changes in renal and oxidative markers, possibly through its free radical scavenging activity. Thus, OMEE may be combined with cisplatin to alleviate nephrotoxicity in cancer chemotherapy.

## 1. Introduction

Many anticancer drugs have teratogenic and other serious effects on biological systems prompting restricted usage [1]. *Cis*-Diaminedichloroplatinum (II) (CDDP), typically known as cisplatin, is a heavy metal complex that is one of the most effective anti-neoplastic drugs currently available [2]. However, dose-dependent and cumulative nephrotoxicity is a major side effect of CDDP, leading to a reduction in dosage over time, or ceasing its use despite its potency against a variety of tumor types [3]. Approximately 25%–35% of patients suffer from nephrotoxicity following a single dose of cisplatin [4]. Generally, the kidney is one of the most susceptible target organs for drug-associated toxicity due to its high perfusion rate and high capability for drug uptake and metabolism [5]. El-sayed *et al.* [6] and Ognjanović *et al.* [7] elucidated the cause of nephrotoxicity emerging after CDDP therapy. They reported that cisplatin was preferentially taken up and that it accumulated in the tubular epithelial cells of the proximal kidney tubule and activated in the kidney to a toxic metabolite by the formation of a platinum-glutathione conjugate, and thereafter to a cysteinyl-glycine-platinum-conjugate. The latter is further transformed to a cysteine conjugate which is a metabolically reactive thiol which acts as a promoter of cellular damage [8]. Further, cisplatin generates reactive oxygen species (ROS), increases lipid peroxidation, and inhibits the activity of antioxidant enzymes in renal tissue which ultimately cause nephrotoxicity [2,9]. Karadeniz *et al.* [10] explained the relationship between CDDP and ROS. ROS such as hydrogen peroxide, hydroxyl radicals, singlet oxygen, superoxide anions, and peroxyl radicals form inside cells by exposure to several endogenous and exogenous agents (CDDP in this case), causing damage to many important biomolecules that have been implicated in several diseases; among them is renal toxicity. These pro-oxidants are kept in balance with endogenous antioxidants, but under disease conditions; the balance is shifted in favor of pro-oxidants leading to oxidative stress (OxS). Excess ROS causes oxidative damage by attacking biomolecules such as membrane lipids, DNA, and proteins in cells [11]. Endogenous antioxidants such as glutathione reduced (GSH), glutathione peroxidase (GSH-PX), superoxide dismutase (SOD), catalase (CAT) are compounds that act as free radical scavengers. These antioxidants are electron donors and can react with free radicals to form harmless products. Thus, the generation of ROS and/or depletion of antioxidant status in renal tissues due to CDDP exposure is one of the causative mechanisms of nephrotoxicity. The clinical usefulness of CDDP has been limited because of its nephrotoxic side effects [12]. Therefore, alleviating the nephrotoxic effect of cisplatin by creating new agents remains a major goal.

As ROS and/or the depletion of antioxidants are crucial factors for triggering nephrotoxicity, natural antioxidants are considered an effective strategy for solving the problem of CDDP toxicity due to their pluripotent activities including antioxidant, antimutagenic, and anticarcinogenic effects [13,14]. Thus, natural antioxidants are preferred as they not just diminish severe side effects that have resulted from the use of anticancer drugs, but also enhance the anticancer activities of antitumor drugs [15]. Many previous studies have revealed that antioxidants of plant origin have an antinephrotoxic effect [16,17,18]. Therefore, in the present study we selected *Origanum majorana* ethanolic extract (OMEE) as it has previously been reported to have antioxidant properties [19]. However, there is minimal data on the effect of *Origanum majorana* against cisplatin-induced nephrotoxicity. Thus, the main purpose of this study was to evaluate the therapeutic potential of OMEE against CDDP-induced nephrotoxicity using an *in vivo* rat model which may consequently be used to alleviate nephrotoxicity in patients undergoing cancer chemotherapy with cisplatin.

## 2. Materials and Methods

### 2.1. Chemicals

Cisplatin (CDDP) was obtained in the form of a Cisplatin Vial (1 mg/mL) (Mylan Institutional LLC, Rockford, IL USA, manufactured by Agila Specialties Pvt. Ltd., Bangalore, India). All kits were purchased from a Bio-diagnostic company for diagnostic and research reagents (Dokki, Giza, Egypt). All other chemicals and solvents were of analytical grade and were purchased from local firms.

### 2.2. Preparation of Origanum Majorana Ethanolic Extract

An ethanolic extract of *Origanum majorana* was prepared by soaking 25 g of *O. majorana* flowers in 150 mL ethanol for 24 h and the filtrate re-soaked for another 24 h (2× extraction). The pooled extracts were concentrated using a rotary evaporator and dried by a lyophilizer (LABCONCO lyophilizer, shell, freeze system, England, UK) at −40 °C, and the extract was stored in a desiccator until use [19]. The dried extract was dissolved in distilled water. The prepared OMEE was used for the investigation of antinephrotoxicity and analyzed for its *in vivo* antioxidant activities.

### 2.3. Animals

Experimental animals were handled according to the Institutional Animal Care and Use Committee (Animal ethics approval number: CUFS/F/PHY/05/13) of the Faculty of Science, Cairo University, Egypt. All experiments were performed with adult male Wistar albino rats weighing approximately 150 ± 5 g. The Wistar rats (*Rattus norvegicus*) were purchased from the animal house of the National Research Center, Egypt. Animals were allowed to acclimatize for 7 days prior to treatment in a 12-h light/dark cycle. They were fed standard laboratory food and provided with water *ad libitum*.

### 2.4. Experimental Design

In order to assess the *in vivo* antinephrotoxic effect of OMEE against cisplatin-induced acute nephrotoxicity, the experimental animals were divided into three groups (*n* = 6/group) and treated as follows:

Group 1: Control group, treated intraperitoneally (i.p.) with a single dose of saline and after three days rats received distilled water orally for 14 days.

Group 2: CDDP group, received a single i.p. dosage of CDDP (3 mg/kg b.wt), then 3 days after the CDDP injection; rats were administered distilled water (Vehicle) for the next 14 days [20].

Group 3: CDDP + OMEE group, rats were injected once with CDDP, followed by oral OMEE administration after 3 days with a dosage of 500 mg/kg b.wt for 14 alternative days. The OMEE dose was selected according to its safety [19].

### 2.5. Animal Handling

On the 18th day, all animals were euthanized under sodium pentobarbital (30 mg/kg b.wt) anaesthesia. Serum was separated from the collected blood and stored at −20 °C for renal marker determination. Kidneys were quickly excised, freed from surrounding fat, and blotted with a piece of filter paper. The right kidney was frozen at −20 °C until used for tissue homogenate preparation, whereas the left kidney was fixed immediately in 10% neutral buffered formalin for light microscopy study.

### 2.6. Tissue Preparation

The right kidney was homogenized (10% w/v) in ice cold 0.1 M Tris-HCl buffer (pH = 7.4) using a Potter Elvehjem homogenizer. The homogenate was centrifuged at 22,000 *g* at 4 °C for 15 min. [21]. The supernatant was collected and used for antioxidant defense enzyme activity assays and for total protein determination.

To evaluate the efficiency of OMEE against CDDP nephrotoxicity, the percentage recovery was calculated to express to what extent the OMEE can attenuate the severity of CDDP nephrooxidative toxicity.

(1) $$\%\;\mathrm{Recovery}=\;\frac{\text{Mean of OMEE }-\text{Mean of CDDP}}{\text{Mean of control}}\;\times 100$$

### 2.7. Evaluation of in VivoAntinephrotoxic Activity of OMEE

The levels of renal function markers included creatinine, urea, and uric acid were determined. Estimates were obtained from serum using commercially available Biodiagnostic kits (Biodiagnostic, Dokki, Giza, Egypt) [22,23,24]. The blood urea nitrogen (BUN) was calculated from the urea concentration by using the factor stated in urea kit.

### 2.8. Evaluation of in Vivo Antioxidant Capacity of OMEE

Malondialdehyde (MDA), glutathione reduced (GSH), catalase (CAT), superoxide dismutase (SOD), and nitric oxide (NO) contents were estimated in kidney supernatant [25,26,27,28,29] using Biodiagnostic kits (Biodiagnostic, Dokki, Giza, Egypt). Total protein was determined in kidney supernatant using Biodiagnostic kit according to the method described by Gornal *et al.* [30] to express the enzymes activity per g protein.

### 2.9. Histological Examination

At the end of the experiment (18 days), animals were euthanized and the left kidney was fixed at 10% neutral buffered formalin solution for histologic examination. Following preparation and staining with hematoxylin-eosin, the slides were examined by a pathologist who was unaware of details of animal groups with light microscopy.

### 2.10. Statistical Analysis

All data are presented as means ± SEM. The statistical significance of the results was evaluated by using a one-way ANOVA test and post-comparison was carried out using a Student’s *t*-test. A probability value less than 0.05 (*p* < 0.05) was considered statistically significant. The statistical package used was statistical package for the social sciences (SPSS, version 15.0, Chicago, IL, USA).

## 3. Results

### 3.1. Effect of OMEE Treatment on the Nephrotoxicity Induced by CDDP

The intense nephrotoxicity model was established during three days following a single injection of CDDP (3 mg/kg b.wt). This manifested in a marked reduction in renal function markers characterized by significant increases (*p* < 0.05) in serum creatinine, urea, uric acid, and BUN levels as compared to the control group. On the contrary, treatment with OMEE markedly reversed cisplatin-induced renal dysfunction by decreasing the creatinine, urea, uric acid, and BUN levels significantly (*p* < 0.05) compared with the CDDP group (Table 1). The present finding revealed the potency of *O. majorana* extract to inhibit the renal toxicity caused by CDDP, as OMEE reduced the creatinine, urea, uric acid, and BUN levels by −57.78%, −55.39%, −42.87%, and −37.67%, respectively as compared with CDDP treatment alone.

### 3.2. In Vivo Antioxidant Potency of OMEE

CDDP (3 mg/kg) produced a significant increase (*p* < 0.05) in MDA and NO contents of renal tissue, as compared with the control group. CDDP diminishes the antioxidant defense system by decreasing the GSH, SOD and CAT renal contents significantly (*p* < 0.05) when compared with the corresponding values of the control group. Post-treatment with OMEE was very effective in the prevention of oxidative damage induced by CDDP, where it lowered the MDA and NO levels significantly (*p* < 0.05) by −107.74% and −73.02%, respectively. Additionally, OMEE significantly potentiated (*p* < 0.05) the renal antioxidant molecules such as GSH, SOD and CAT by 38.56%, 35.42% and 48.42%, respectively, in comparison with the cisplatin-treated group (Table 2).

### 3.3. Histopathology of Rat Kidney

**Figure 1:**
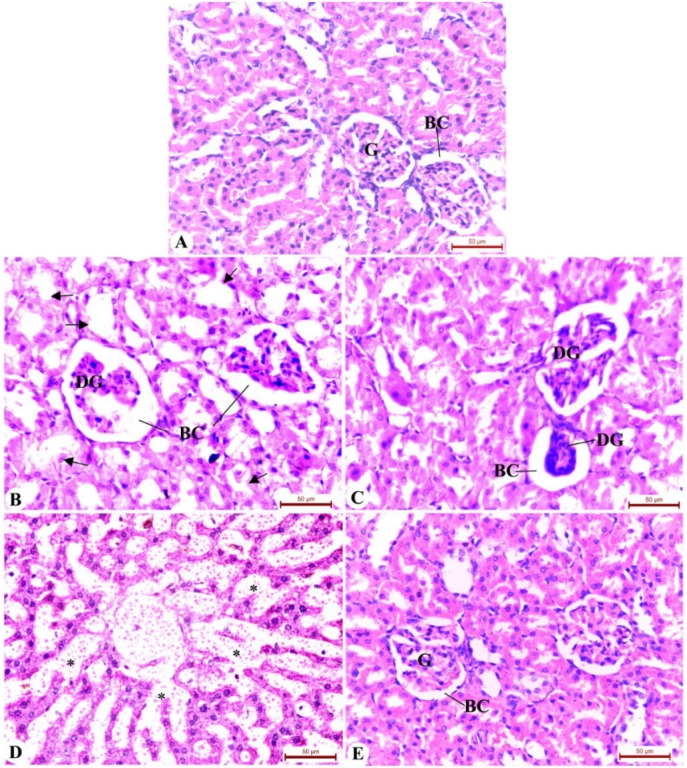
Photomicrographs of kidney of the control and treated groups stained by hematoxylin and eosin. (**A**) Kidney section of control rats showing normal glomeruli (G), Bowman’s space (BC) and normal tubules; ((**B**)–(**D**)) CDDP group showing severe glomerular degeneration (DG), dilatation in Bowman’s space, and degeneration in tubular cells (arrow) in kidney, tubular necrosis invaded by inflammatory cells (*); (**E**) Kidney of post-treatment with the OMEE showing normal renal corpuscle and renal tubule more or less like a normal structure with the regeneration of some renal tubules. OMEE: *Originum majorana* ethanolic extract; CDDP: *Cis*-Diaminedichloroplatinum (II).

The histological changes observed in the kidneys are presented in Figure 1. The kidney of control rats exhibited normal renal tissue, where normal corpuscular and tubular histological structure was observed (Figure 1A). The cisplatin-exposed animals showed distinctive pathologic alterations such as degenerated glomeruli, glomerular atrophy, dilatation in Bowman’s space, tubular degeneration, luminal dilatation, and tubular necrosis with invading inflammatory cells (Figure 1B–D). Evidence from numerous sections indicates that the above mentioned changes were extensive and nephrons were observed throughout. Post-treatment with OMEE dramatically reduced cisplatin nephrotoxicity as evidenced by the fact that renal corpuscles and tubules appeared to be similar to those of the control group (Figure 1E). Evidence from the histological study supports the biochemical analyses and ascertainthat OMEE has antinephrotoxic activity.

## 4. Discussion

CDDP is one of the most active drugs used to treat a variety of cancers. Unfortunately, CDDP preferentially accumulates in the kidney to a greater degree than in other organs. This contributes to CDDP nephrotoxicity and hence restricts its clinical utilization for long term treatment [31]. In addition, CDDP may cause acute renal failure even after a single dose [32]. Thus, it is important to discover nontoxic agents that can diminish nephrotoxicity induced by CDDP chemotherapy. Therefore, in the present study, a quantitative evaluation of CDDP-induced structural functional alterations in the kidneys was performed by histopathological and biochemical analyses in order to determine the potential beneficial effects of OMEE on CDDP-induced nephrotoxicity.

The present study revealed that a single injection of 3 mg CDDP/kg b.wt was sufficient to induce nephrotoxicity in rats as serum creatinine, urea, uric acid, and blood urea nitrogen levels were elevated. These results are consistent with those reported by Danduga *et al.* [18] and Palipoch and Punsawad [33]. CDDP elicits OxS in the mitochondria of kidney proximal tubular and endothelial cells, followed by generation of a secondary wave of ROS/RNS (reactive oxygen/nitrogen species), deterioration of mitochondrial structure and function, and eventually renal damage [34]. Moreover, the present study showed that an injection of CDDP induced renal oxidative damage after just three days. This was manifested by a marked increase in renal MDA (a secondary product of lipid peroxidation), and NO levels, and depletion of the renal antioxidant molecules such as GSH, SOD, and CAT contents. These findings are in line with the findings of Ognjanović *et al.* [7]; Karadeniz *et al.* [10]; and Danduga *et al.* [18]. The current study hypothesized that the rise of renal markers in serum is due to the generation of ROS and/or depletion of antioxidant molecules. As the CDDP accumulated in the kidney tissue, it covalently bound with the kidney proteins which in turn impact antioxidant enzymes. In this way, tissue damage occurs and renal markers are released into the circulatory system. The present histological damage of renal tissue following treatment with CDDP strengthens this hypothesis as rats exposed to CDDP revealed severe glomerular and tubular degeneration. From the overall results, we propose that the sequence of renal damage induced by CDDP occurred as follows: CDDP initially evoked oxidative renal damage followed by deterioration in its morphological architecture which eventually led to liberation of tissue markers in the blood. This hypothesis is in agreement with the suggestion of El-Gerbed [2]. Ognjanović *et al.* [7] clarified that CDDP triggers free radical production, which is responsible for oxidative renal damage. The generated free radicals interact with the membrane lipids causing their peroxidation. This notion is supported by the increase MDA level, consequently affecting the cellular structure and function. We regard this is the etiology of kidney dysfunction/nephropathy induced by CDDP.

In the current study, we speculated that the generation of ROS and the inhibition of thioredoxin reductase (TrxR) occurred in response to treatment with CDDP. The thioredoxin system plays an important role in the regulation of antioxidant defense and redox status of the cells [35]. TrxR is a critical component of the mammalian thioredoxin system and is overexpressed in many cancer cells. CDDP inhibits this system and hence may raise the OxS status due to an imbalance of antioxidant/oxidant molecules [36]. Furthermore, it has been shown that high NO level exert toxicological effects by reacting with superoxide anions (O^2−^) to generate short-lived but hyperactive peroxynitrite radicals (ONOO^−^) with subsequent nitration of protein tyrosine residues [37]. Also, NO output depletes intracellular GSH which increases susceptibility to OxS and aggravates renal tissue damage, especially in glomerular diseases [38]. Prabhu *et al.* [34] clarified that the decreased SOD level in CDDP treated animals may be due to the depletion of copper and zinc in the kidney, which are essential for the activity of enzymes. In addition, Peres and da Cunha [39] disclosed that cisplatin penetrates the tubular cells of the kidneys and reaches high concentration in the proximal tubules (S3 segment). Tubular damage assessed through impaired reabsorption, which is characterized by reduced glomerular filtration rate, increased serum creatinine and blood urea concentrations.

Complementary natural therapies have been recently combined with chemotherapies to reduce the adverse effects of the latter as many natural therapies are considered chemoprotective agents [7,40]. Biochemical and histological results of the present study revealed that OMEE attenuate nephrotoxicity induced by CDDP, since a marked recovery was observed in the renal function markers of CDDP rats following treatment with OMEE. In the present study, serum creatinine, urea, uric acid, and BUN levels returned approximately to the normal control levels when animals were administered OMEE subsequent to CDDP treatment (Table 1). Further, OMEE may regenerate renal corpuscle and tubules as observed in the histological architecture (Figure 1). This indicates that OMEE has therapeutic potential on CDDP-induced nephrotoxicity. The present study assumed that the anti-nephrotic effect of OMEE is due to its free radicals and antioxidant capacity [19]. Desouky *et al.* [19] verified the antioxidant activity of OMEE through its inhibition to the DPPH (2, 2-diphenyl-1-picrylhydrazyl) free radical. This free radical inhibiting activity suggests electron donating activity and reductive potential of the OMEE. Thereby, OMEE may inhibit lipid peroxidation and increase intracellular concentration of glutathione and other antioxidant enzymes. Furthermore, it was reported that antioxidants can reduce certain types of toxicity associated with chemotherapy [41,42]. This assumption was verified through the present *in vivo* study wherein the antioxidant potency of OMEE was demonstrated. It is clear that OMEE can promote renal antioxidant status and/or diminish ROS of the CDDP-nephrotoxic rats. This was evidenced by the significant decrease in MDA, NO levels and a significant increase in each of GSH, SOD, and CAT renal contents (Table 2). The antioxidative and consequently the antinephrotoxic activities of the OMEE may be due to its various secondary metabolites such as flavonoids, tannins, sterols, triterpens, glycosides, alkaloids, and saponins which were confirmed in a previous investigation [19]. It has also been reported that flavonoids in plant extract have a repairing effect on damaged organ tissue and prevent oxidative cell damage via their water soluble properties [43]. Moreover, the presence of these secondary metabolites in the OMEE may have therapeutic activity against several diseases and this supports its traditional use for the treatment of various illnesses such as nephrotoxicity. The beneficial role of OMEE together with its safety profile [19] enables the OMEE to be tested and used to reverse CDDP-induced nephrotoxicity without any adverse effect.

## 5. Conclusions

CDDP-induced nephrotoxicity in rats was established after a single injection (3 mg/kg b.wt). However, the present study revealed that treatment with an *Originum majorana* ethanolic extract (OMEE) reversed the nephrotoxicity caused by CDDP. Thus, this study provides evidence regarding the promising antinephrotoxic effect of OMEE against CDDP toxicity. The therapeutic effect of OMEE may be mediated via its antioxidant effect. Hence, the present work opens a window to a novel therapy to manage CDDP-induced nephrotoxicity in cancer patients undergoing chemotherapy with CDDP that may enhance the quality of life and prolong survival. The present findings are considered preliminary with respect to the antinephrotoxic activity of OMEE against CDDP; further investigations aimed at the isolation of compounds are required to identify additional mechanisms of action of OMEE as the OxS is not the only pathway of cisplatin toxicity. Furthermore, additional clinical trials in cancer patients receiving CPPD therapy are required to establish this hypothesis.

## Figures and Tables

**Table 1:** Effect of *Originum majorana* ethanolic extract (OMEE) on *Cis*-Diaminedichloroplatinum (II) (CDDP)-induced nephrotoxicity.

Markers		Control	CDDP		
			Vehicle	OMEE	
Creatinine	(mg/dL)	0.448 ± 0.013	0.727 ± 0.047 ^a^	0.465 ± 0.027 ^b^	(−57.78%)
Urea	(g/dL)	38.145 ± 1.065	54.598 ± 4.042 ^a^	33.466 ± 1.451 ^b^	(−42.87%)
Uric acid	(mg/dL)	2.148 ± 0.081	2.742 ± 0.165 ^a^	1.926 ± 0.212 ^b^	(−42.87%)
BUN	(g/dL)	81.718 ± 2.278	116.911 ± 8.654 ^a^	81.879± 6.303 ^b^	(−37.67%)

All data are mean ± SEM of 6 rats; ^a^: significant at *p* < 0.05 as compared to control group; ^b^: significant at *p* < 0.05 as compared to CDDP group; (%) is the % improvement of each treatment against CDDP. BUN = blood urea nitrogen; OMEE = *Originum majorana* ethanolic extract; CDDP = *Cis*-Diaminedichloroplatinum (II).

**Table 2:** Effect of OMEE on CDDP-induced oxidative/nitrosative damage.

Markers		Control	CDDP		
			Vehicle	OMEE	
MDA	(nmol/g.protein)	5.937 ± 0.595	12.029 ± 0.711 ^a^	5.628 ± 0.527 ^b^	(−107.74%)
NO	(Mmol/g.protein)	171.602 ± 9.944	312.555 ± 19.110 ^a^	187.258 ± 24.846 ^b^	(−73.02%)
GSH	(mg/g.protein)	22.719 ± 2.885	13.621 ± 2.098 ^a^	22.378 ± 1.591 ^b^	(38.56%)
SOD	(U/g.protein)	993.255 ± 74.932	655.220 ± 87.654 ^a^	991.462 ± 105.168 ^b^	(35.42%)
CAT	(U/g.protein)	11.054 ± 0.502	5.730 ± 0.776 ^a^	11.081 ± 1.161 ^b^	(48.42%)

All data are mean ± SEM of 6 rats; ^a^: significant at *p* < 0.05 as compared to control group; ^b^: significant at *p* < 0.05 as compared to CDDP group; (%) is the % improvement of each treatment against CDDP. OMEE: *Originum majorana* ethanolic extract; CDDP: *Cis*-Diaminedichloroplatinum (II); MDA: Malondialdehyde; NO: Nitric oxide; GSH: Glutathione reduced; SOD: Superoxide dismutase; CAT: Catalase.
